# Author Correction: Comparative genomics reveal a novel phylotaxonomic order in the genus *Fusobacterium*

**DOI:** 10.1038/s42003-025-07578-y

**Published:** 2025-02-01

**Authors:** Cristian Molteni, Diego Forni, Rachele Cagliani, Manuela Sironi

**Affiliations:** Scientific Institute IRCCS E. MEDEA, Bioinformatics, Bosisio Parini, Italy

**Keywords:** Bacteria, Population genetics

Correction to: *Communications Biology* 10.1038/s42003-024-06825-y, published online 07 September 2024

In the original version of the article, the second paragraph of the section “Genetic relationships in the *F. nucleatum*/*F. periodonticum* lineage”, contained the statement that “Indeed, one of these is Fusobacterium FNU, previously suggested to represent a new species.” This has now been corrected to “Indeed, one of these is Fusobacterium FNU, previously suggested to represent a new subspecies.”

Further, the statement “Thus, evidence herein calls for a re-analysis of *F. nucleatum animalis* features associated to CRC.” in the abstract is now phrased “Thus, evidence herein calls for further evolutionary and phylogenomic analyses based on more *Flavobacterium nucleatum* genome sequences.”

Lastly, the following sentence in the discussion, “All these differences would be noteworthy for bacteria in the same species, but we show here that this is not the case. Indeed, all these features were compared between two distinct species, although closely related. Whereas this is unlikely to change the conclusion that *F. nucleatum* *animalis* is associated with CRC, our data call for a re-assessment of the characteristics of clade C2, which were established in comparison to a different species.” has been replaced by “All these differences are noteworthy, but because our data suggest a different phylotaxonomic arrangement, future phylogenomic re-analyses of the characteristics of the new species/clade C2 is desirable.”

