# Supplementary material for: Comparative genomics reveal a novel phylotaxonomic order in the genus Fusobacterium
Source: Commun Biol. 2024 Sep 7;7:1102. doi: 10.1038/s42003-024-06825-y (PMC11380691; doi:10.1038/s42003-024-06825-y)
Supplement: Supplementary file 1 — Supplementary Information [file 42003_2024_6825_MOESM1_ESM.pdf]

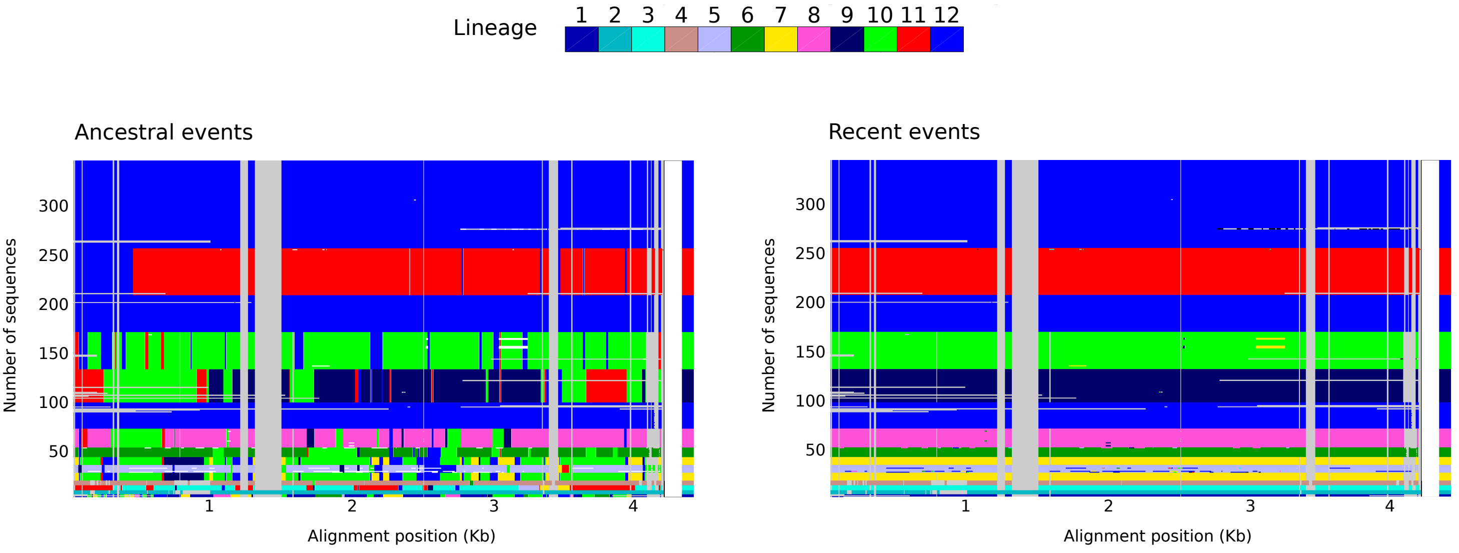

Supplementary Figure 1. Plot of fastGEAR results on the rpoB alignment. Ancestral recombination events are plotted on the left, recent ones on the right. Recombination between lineages is depicted with different colors. Gray columns represents gaps in the alignment. For the ancestral recombination plot, recent recombination events are shown as white gaps.

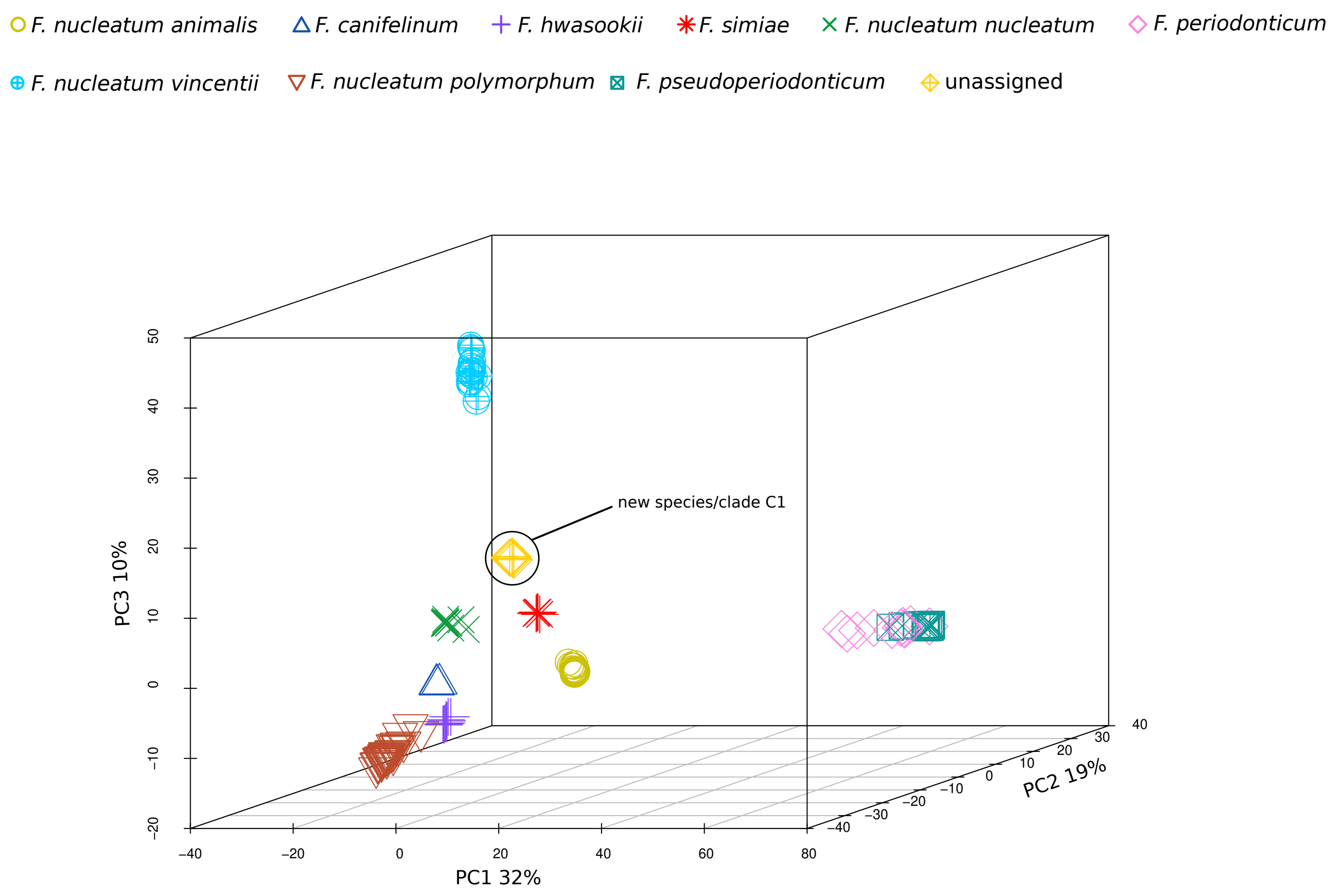

Supplementary Figure 2. Three components PCA plot. 3D representation of the first three components calculated using core genes. Symbols and colors are listed in the legend. See also Figure 6A.

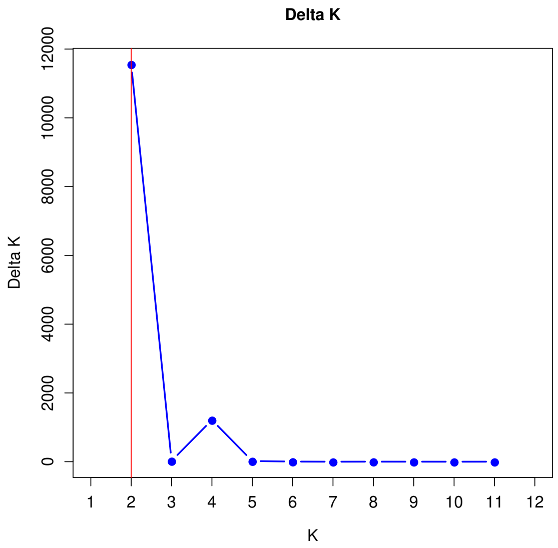

Supplementary Figure 3. Analysis of optimal K for the STRUCTURE no admixture model. ΔK is calculated as ΔK = mean(|L''(K)|) / sd(L(K)). The peaks of the plotted line are the optimal K used in STRUCTURE analysis.

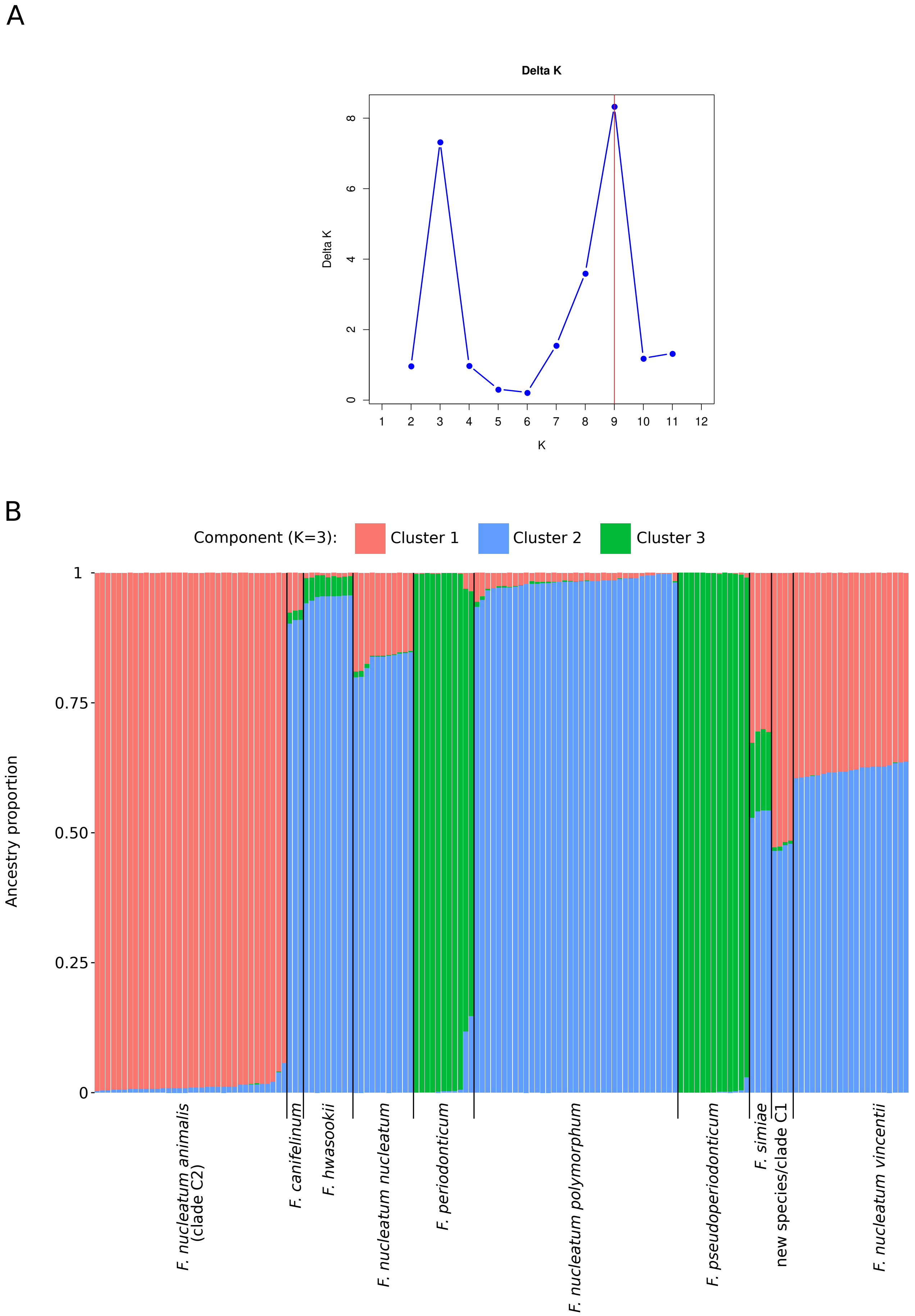

Supplementary Figure 4. Analysis of STRUCTURE linkage model. (A) Evanno's method plot for optimal K. ΔK is calculated as ΔK = mean(|L''(K)|) / sd(L(K)). The peaks indicate the optimal K selected for STRUCTURE analysis. (B) Bar plot representing the proportion of ancestral population components for K=3. Each vertical line represents a Fusobacterium core genome and it is colored by the proportion of sites that have been assigned to one of the populations by STRUCTURE.

Supplementary Table 1. List of recombination events identified by fastGEAR.

Gene	Number of Strains	Number of Recent Events	Number of Ancestral Events	KEGG orthology
prfA	348	14	37	K02835; peptide chain release factor 1
prfB	345	50	93	K02836; peptide chain release factor 2
rseP	352	70	59	K11749; regulator of sigma E protease
ftsZ	346	53	49	K03531; cell division protein FtsZ
ychF	358	36	73	K06942; ribosome-binding ATPase
tig	350	99	91	K03545; trigger factor
uvrC	342	88	231	K03703; excinuclease ABC subunit C
alaS	355	67	143	K01872, alaS; alanyl-tRNA synthetase
ileS	312	70	135	K01870; isoleucyl-tRNA synthetase
leuS	353	192	153	K01869; leucyl-tRNA synthetase
metG	348	49	192	K01874; methionyl-tRNA synthetase
serS	355	33	89	K01875; seryl-tRNA synthetase
radA	355	135	109	K04485; DNA repair protein RadA/Sms
cysS	352	28	152	K01883; cysteinyl-tRNA synthetase
hisS	351	38	92	K01892; histidyl-tRNA synthetase
argS	346	65	107	K01887; arginyl-tRNA synthetase
aspS	354	69	118	K01876; aspartyl-tRNA synthetase
pheT	350	113	215	K01890; phenylalanyl-tRNA synthetase beta chain
infB	355	41	246	K02519; translation initiation factor IF-2
hemN	338	69	148	K02495; oxygen-independent coproporphyrinogen III oxidase Enzymes
mfd	355	400	446	K03723; transcription-repair coupling factor (superfamily II helicase)
polA	338	374	405	K02320; DNA polymerase alpha subunit A
uvrB	353	164	259	K03702; excinuclease ABC subunit B
recN	347	159	114	K03631; DNA repair protein RecN (Recombination protein N)
recG	354	190	286	K03655; ATP-dependent DNA helicase RecG
rpsA	345	103	62	K02945; small subunit ribosomal protein S1
purB	347	59	42	K01756; adenylosuccinate lyase
ffh	348	53	80	K00973; glucose-1-phosphate thymidylyltransferase
secA	348	122	245	K03070; preprotein translocase subunit

				SecA
secY	342	12	40	K03076; preprotein translocase subunit SecY
atpD	348	31	66	K02112; F-type H+/Na+-transporting ATPase subunit beta
gyrB	341	79	37	K02470; DNA gyrase subunit B
gyrA	346	92	64	K02469; DNA gyrase subunit A
murC	352	71	88	K01924; UDP-N-acetylmuramate--alanine ligase
murD	352	165	174	K01925; UDP-N-acetylmuramoylalanine--D-glutamate ligase
guaB	349	21	51	K00088; IMP dehydrogenase
dnaG	343	183	195	K02316; DNA primase
lepA	343	28	67	K03596; GTP-binding protein LepA
typA	349	40	52	K06207; GTP-binding protein
nusA	352	52	76	K02600; transcription termination/antitermination protein NusA
rpoB	345	98	198	K03043; DNA-directed RNA polymerase subunit beta
dnaK	350	26	60	K04043; molecular chaperone DnaK
rpoC	348	94	235	K03046; DNA-directed RNA polymerase subunit beta'
dnaX	346	298	187	K02343; DNA polymerase III subunit gamma/tau
der	346	32	71	FN0170; GTP-binding protein

Supplementary Table 2. Abundance of different *Fusobacterium* species in different oral sites.

	Sub-gingival Plaque	Supra-gingival Plaque	Keratinized Gingiva	Buccal Mucosa	Hard Palate	Saliva	Throat	Palatine Tonsils	Tongue Dorsum
F. Periodonticum and *F. Pseudoperiodonticum*									
Avg Eren et al.(2014) (V1-V3)	0.064	0.062	0.048	0.264	0.529	0.669	0.965	1.23	1.422
Avg Eren et al.(2014) (V3-V5)	0.308	0.393	0.397	1.694	3.031	4.947	4.979	6.947	8.126
Avg Dewhirst 35x9 data (not published yet)	0.085	0.273	0.851	0.864	1.124	2.81	3.057	2.423	2.775
**AVERAGE**	**0.15**	**0.24**	**0.43**	**0.94**	**1.56**	**2.81**	**3**	**3.53**	**4.11**
**STANDARD DEVIATION**	**0.14**	**0.17**	**0.4**	**0.72**	**1.31**	**2.14**	**2.01**	**3.02**	**3.55**
F. nucleatum (all subspecies) and *F. hwasookii*									
Avg Eren et al.(2014) (V1-V3)	6.019	2.326	0.586	0.51	0.129	0.659	0.73	2.588	0.083
Avg Eren et al.(2014) (V3-V5)	10.356	5.39	0.832	0.917	0.243	1.358	0.788	2.974	0.206
Avg Dewhirst 35x9 data (not published yet)	12.027	8.268	0.981	1.517	0.98	0.365	0.626	2.607	0.128
**AVERAGE**	**9.47**	**5.33**	**0.8**	**0.98**	**0.45**	**0.79**	**0.71**	**2.72**	**0.14**
**STANDARD DEVIATION**	**3.1**	**2.97**	**0.2**	**0.51**	**0.46**	**0.51**	**0.08**	**0.22**	**0.06**
