# Supplementary material for: Comparative genomics reveal a novel phylotaxonomic order in the genus Fusobacterium
Source: Commun Biol. 2024 Sep 7;7:1102. doi: 10.1038/s42003-024-06825-y (PMC11380691; doi:10.1038/s42003-024-06825-y)
Supplement: Supplementary file 2 — Description of Additional Supplementary File [file 42003_2024_6825_MOESM2_ESM.pdf]

Description of Additional Supplementary Files

File name: Supplementary Data 1

Description: list of strains analyzed in this work
