# Supplementary material for: Comparative genomics reveal a novel phylotaxonomic order in the genus Fusobacterium
Source: Commun Biol. 2024 Sep 7;7:1102. doi: 10.1038/s42003-024-06825-y (PMC11380691; doi:10.1038/s42003-024-06825-y)
Supplement: Supplementary file 4 — reporting summary [file 42003_2024_6825_MOESM4_ESM.pdf]

nature portfolio

Corresponding author(s): Cristian Molteni

Last updated by author(s): Aug 30, 2024

Reporting Summary

Nature Portfolio wishes to improve the reproducibility of the work that we publish. This form provides structure for consistency and transparency in reporting. For further information on Nature Portfolio policies, see our Editorial Policies and the Editorial Policy Checklist.

Statistics

For all statistical analyses, confirm that the following items are present in the figure legend, table legend, main text, or Methods section.

n/a	Confirmed	
☒	☐	The exact sample size (*n*) for each experimental group/condition, given as a discrete number and unit of measurement
☒	☐	A statement on whether measurements were taken from distinct samples or whether the same sample was measured repeatedly
☒	☐	The statistical test(s) used AND whether they are one- or two-sided<br>*Only common tests should be described solely by name; describe more complex techniques in the Methods section.*
☒	☐	A description of all covariates tested
☒	☐	A description of any assumptions or corrections, such as tests of normality and adjustment for multiple comparisons
☒	☐	A full description of the statistical parameters including central tendency (e.g. means) or other basic estimates (e.g. regression coefficient) AND variation (e.g. standard deviation) or associated estimates of uncertainty (e.g. confidence intervals)
☒	☐	For null hypothesis testing, the test statistic (e.g. *F*, *t*, *r*) with confidence intervals, effect sizes, degrees of freedom and *P* value noted<br>*Give P values as exact values whenever suitable.*
☐	☒	For Bayesian analysis, information on the choice of priors and Markov chain Monte Carlo settings
☒	☐	For hierarchical and complex designs, identification of the appropriate level for tests and full reporting of outcomes
☒	☐	Estimates of effect sizes (e.g. Cohen's *d*, Pearson's *r*), indicating how they were calculated

Our web collection on statistics for biologists contains articles on many of the points above.

Software and code

Policy information about availability of computer code

Data collection	Genome sequences were downloaded with the getGenome function from the R package biomaRt.
Data analysis	Complete and draft genome sequences were obtained from the NCBI database by using the getGenome function from the R package biomaRt; genomes were used as input data for the Genome Taxonomy Database Toolkit v2.1.1 to identify marker genes. alignments were constructed using MAFFT v7.475. A neighbor-net split network was generated throughout SplitsTree4 and recombination was evaluated with fastGEAR and Gubbins. Sequence similarity analyses were performed using SimPlot++ v1.3 and pyANI. Pangenomes were analyzed with PATO. Principal component analysis was performed with the mixOmics R package, and population structure was analyzed with the STRUCTURE software v2.3.4. Nucleotide diversity and Tajima's D were calculated using the DnaSP software v61203.

For manuscripts utilizing custom algorithms or software that are central to the research but not yet described in published literature, software must be made available to editors and reviewers. We strongly encourage code deposition in a community repository (e.g. GitHub). See the Nature Portfolio guidelines for submitting code & software for further information.

Data

Policy information about availability of data

All manuscripts must include a data availability statement. This statement should provide the following information, where applicable:

- Accession codes, unique identifiers, or web links for publicly available datasets
- A description of any restrictions on data availability
- For clinical datasets or third party data, please ensure that the statement adheres to our policy

The list of genome IDs is reported is Supplementary Data 1.

Research involving human participants, their data, or biological material

Policy information about studies with human participants or human data. See also policy information about sex, gender (identity/presentation), and sexual orientation and race, ethnicity and racism.

Reporting on sex and gender	Not applicable.
Reporting on race, ethnicity, or other socially relevant groupings	Not applicable.
Population characteristics	Not applicable.
Recruitment	Not applicable.
Ethics oversight	Not applicable.

Note that full information on the approval of the study protocol must also be provided in the manuscript.

Field-specific reporting

Please select the one below that is the best fit for your research. If you are not sure, read the appropriate sections before making your selection.

☐ Life sciences ☐ Behavioural & social sciences ☒ Ecological, evolutionary & environmental sciences

For a reference copy of the document with all sections, see nature.com/documents/nr-reporting-summary-flat.pdf

Ecological, evolutionary & environmental sciences study design

All studies must disclose on these points even when the disclosure is negative.

Study description	This study applies comprehensive analyses to investigate the genetic diversity and relationships within the Fusobacterium genus and also to identify miss-classified and undescribed species.
Research sample	The research sample is composed by a set of 361 Fusobacterium genomes that represent the genetic diversity among the genus. The list of Fusobacterium genomes was derived from the BV-BRC site. Complete and draft genome sequences were obtained from the NCBI database.
Sampling strategy	All available complete/near complete genomes were included in the study.
Data collection	Data were collected from public curated databases, selecting high quality genomes.
Timing and spatial scale	The data in this study is based on multiple reports from several geographical and temporal collections.
Data exclusions	no data were excluded from the analysis.
Reproducibility	Population structure analyses were repeated ten times for each K population.
Randomization	Strains were grouped based on different genomics and phylogenetic tools. Strains that were miss-classified in previous analyses were reported and fixed.
Blinding	All strains were considered unclassified and reclassified in this study.

Did the study involve field work? ☐ Yes ☒ No

Reporting for specific materials, systems and methods

We require information from authors about some types of materials, experimental systems and methods used in many studies. Here, indicate whether each material, system or method listed is relevant to your study. If you are not sure if a list item applies to your research, read the appropriate section before selecting a response.

Materials & experimental systems

n/a	Involved in the study
☒	☐ Antibodies
☒	☐ Eukaryotic cell lines
☒	☐ Palaeontology and archaeology
☒	☐ Animals and other organisms
☒	☐ Clinical data
☒	☐ Dual use research of concern
☒	☐ Plants

Methods

n/a	Involved in the study
☒	☐ ChIP-seq
☒	☐ Flow cytometry
☒	☐ MRI-based neuroimaging

Plants

Seed stocks

Report on the source of all seed stocks or other plant material used. If applicable, state the seed stock centre and catalogue number. If plant specimens were collected from the field, describe the collection location, date and sampling procedures.

Novel plant genotypes

Describe the methods by which all novel plant genotypes were produced. This includes those generated by transgenic approaches, gene editing, chemical/radiation-based mutagenesis and hybridization. For transgenic lines, describe the transformation method, the number of independent lines analyzed and the generation upon which experiments were performed. For gene-edited lines, describe the editor used, the endogenous sequence targeted for editing, the targeting guide RNA sequence (if applicable) and how the editor was applied.

Authentication

Describe any authentication procedures for each seed stock used or novel genotype generated. Describe any experiments used to assess the effect of a mutation and, where applicable, how potential secondary effects (e.g. second site T-DNA insertions, mosiacism, off-target gene editing) were examined.
